# Role of psychiatrists in early diagnosis and treatment of OCD

**DOI:** 10.1192/bjb.2024.126

**Published:** 2025-02

**Authors:** Ilenia Pampaloni

**Affiliations:** consultant psychiatrist, National OCD and BDD Service, South West London and St George's Mental Health Trust, UK. Email: Ilenia.Pampaloni@swlstg.nhs.uk

Obsessive–compulsive disorder (OCD) is a common, highly disabling and pervasive but treatable early-onset condition. It has been reported to be ranked by the WHO among the top ten most disabling of all disorders, with two-thirds of patients suffering severe impairment.^1^ Nevertheless, it is characterised by a prolonged duration of untreated illness, averaging 9–10 years; this ranks among the longest for any mental disorder^2^ and is correlated with poorer response to selective serotonin reuptake inhibitors,^2^ less frequent remission, higher psychiatric and medical comorbidity, higher disability and greater severity.^3^

This extensive duration of untreated illness can be attributed to various factors, including cultural stigma and misconceptions, particularly towards OCD phenotypes with sexual, religious or aggressive content, poor insight, or avoidance of medical and psychiatric consultations (e.g. for fear of contamination).^3^ There is also a critical need to avoid premature discharge of patients with OCD for non-engagement in assessment and treatment, owing to the avoidance behaviours typical of OCD being overlooked.

There is also an average delay of 2 years from the time of diagnosis before appropriate treatment begins.^2^ This delay includes the time taken to access effective therapies such as cognitive–behavioural therapy (CBT) with exposure and response prevention (ERP) or to start appropriate pharmacological treatments. The lack of immediate access to these treatments contributes significantly to the progression and entrenchment of the disorder.

From a pharmacological perspective, it is essential to adhere to evidence-based treatment recommendations, prescribing selective serotonin reuptake inhibitors at adequate dosages (usually the maximum tolerated *British National Formulary* doses) for sufficient durations and taking into account that full therapeutic effects might not be evident until 12 weeks after reaching the maximum dosage. This is an important intervention to be carried out while waiting for CBT, as it has been shown that combining CBT and pharmacotherapy has the best evidence.^4^

While patients await CBT with ERP, there are important proactive steps that psychiatrists can take. For example, initiating basic CBT formulations highlighting how carrying out compulsions maintains OCD, as well as providing basic concepts about resisting compulsions and explaining how ERP works, can be an effective early intervention while preparing patients for CBT.

Moreover, guiding patients towards self-help resources such as bibliotherapy, peer-led support groups and digital interventions while they are waiting for therapy has shown benefits. Recent randomised controlled trials indicate that bibliotherapy^5^ and internet-based therapies^5^ can be superior to traditional wait-list controls and treatment as usual, offering accessible and effective alternatives during interim periods.^5^

As psychiatrists, enhancing our understanding of and ability to manage OCD from onset is crucial. Our role in providing basic CBT skills, as well as directing patients to accessible resources such as books and digital interventions, is of vital importance. Enhancing training in CBT for psychiatrists to encompass these critical areas could improve our capacity to manage OCD effectively and may also have a significant impact on the early stages of treatment, potentially reducing the overall burden of the disorder and markedly improving patient outcomes and quality of life.
